# Social Media in Health Studies: A Systematic Review of Comparative Learning Methods

**DOI:** 10.3390/ijerph19042205

**Published:** 2022-02-15

**Authors:** Alban Fouasson-Chailloux, Pauline Daley, Pierre Menu, Raphael Gross, Marc Dauty

**Affiliations:** 1 CHU Nantes, Service de Médecine Physique et Réadapatation Locomotrice et Respi-Ratoire, 44093 Nantes, France; pauline.daley@chu-nantes.fr (P.D.); pierre.menu@chu-nantes.fr (P.M.); marc.dauty@chu-nantes.fr (M.D.); 2 CHU Nantes, Service de Médecine du Sport, 44093 Nantes, France; 3 IRMS, Institut Régional de Médecine du Sport, 44093 Nantes, France; 4 Inserm, UMR 1229, Regenerative Medicine and Skeleton, Université de Nantes, 44042 Nantes, France; 5 MIP, EA4334, Laboratoire Motricité-Interactions-Performance, CHU Nantes, Nantes Université, 44000 Nantes, France; raphael.gross@chu-nantes.fr

**Keywords:** social media, students, learning, teaching, health

## Abstract

Learning modifications particularly increased due to the SARS-CoV-2 pandemic, which made it necessary to offer distance health education for many months. Social media allows students to have interactive activities such as discussing specific subjects or sharing data with each other, and also to have interactions with their teachers and tutors. So, we aimed to compare the effects of the use of social media on knowledge, skills and perceptions in health students compared to other methods. We performed a systematic review on PubMed, ScienceDirect and Embase about comparative learning methods using social media. The search followed PRISMA guidelines, and the quality assessment of the studies was performed using the Medical Education Research Quality instrument (MERSQI). Eight studies were analyzed including 1014 participants. Mean age ranged from 19.9 to 23.4 years, and 70% were females. About 54.4% of the participants were medical students and 20.9% were dental students. The mean MERSQI was 11.7 ± 2.6. Various subjects were included—anatomy, cultural competences, sterile surgical techniques, radiology, arthrocentesis, medical pathologies and cariology. As far as knowledge evaluation was concerned, we found that the use of social media may have had a positive effect from a short-term point of view but results concerning skills were less consistent across studies. Students usually had a positive perception of the use of social media as a complementary method but not as a complete alternative so it is not excluded that this effect might result from an increase in working time. The impact on patient care should also be assessed in future studies.

## 1. Introduction

Health studies have undergone many changes in recent decades. Indeed, passive learning in the classroom or amphitheater tends to be supplemented by new teaching and learning approaches [1]. Indeed, these new methods may include video sessions, flipped classrooms or virtual classrooms and e-learning [2,3,4,5]. They seem to provide interesting results in terms of skills and knowledge improvement, but some studies may be more moderate in their results [6,7]. Recently, these learning modifications have particularly increased due to the SARS-CoV-2 pandemic, which has made it necessary to offer distance education for many months, including in clinical fields [2,8,9]. Yet, online classrooms do not show systematically positive results, especially with students, who think it has a negative impact on knowledge, on relationships with teachers and on attentiveness [7]. This might be mainly due to the reduction of teacher–student interaction and student–student interaction. Furthermore, in the last two decades the use of social media has developed in the entire population and especially in young people [10,11], but also among health professionals and students [12,13,14,15]. Social media are digital spaces created for people, providing an environment that is conducive to interaction [16]. It allows professionals and students to share valuable information and content or to participate in networking [17] in order to improve daily practices and knowledge. Moreover, social media enables students to have more interactive activities such as discussing specific subjects or sharing data with each other, but also to have interactions with their teachers and tutors [15,18]. Yet, the evaluation of the use of social media in health education is difficult since a comparative group is lacking in many studies, which limits their scope. Nevertheless, some works comparing different interventions have been published, providing a good level of proof. 

In this systematic review we aimed to assess the impact of the use of social media on health students concerning their capacity to acquire knowledge and skills, but also their perception on this new learning approach compared to traditional learning. We hypothesized that social media might have a positive effect on learning.

## 2. Materials and Methods

### 2.1. Literature Search 

We searched articles in the medical databases: PubMed, ScienceDirect and Embase in November 2021. Article research extended from January 2000 to November 2021. We included only studies in the English language. Searches used the following MeSH: (“Social media” OR “Social network”) AND (“Student” OR “learning” OR “teaching”) AND (“medical” OR “dental” OR “nurse” OR “midwife”). The search was performed independently by two authors (AFC and PD) to assess titles and abstracts of potentially relevant articles, and then the full-text articles were analyzed. In case of disagreement, a third assessor was engaged (PM or MD). All relevant articles were read in full text by the two researchers (AFC, PD) to assess if the articles met the inclusion criteria. Taking the PubMed database as an example, the search strategy is shown in Table 1.

### 2.2. Eligibility Criteria

We included studies assessing the use of social-media learning in health students: medical, dental, midwifery, physical therapy and nurse studies. Studies had to compare at least two types of learning method: social media vs. a traditional method. For techniques classified as traditional method, we considered techniques using classroom or workshop teaching, where the teacher was the controller of the learning environment and, considered the unique source of knowledge [19]. Exclusion criteria included absence of comparative methods or participants who were not health students, or comparisons between two social-media learning methods or two traditional methods. All types of social media and all types of health subjects were considered.

### 2.3. Data Extraction

After analyzing the included studies, relevant data were summarized in tables using Microsoft Excel (version 2013, Microsoft Corporation, Redmond, WA, USA): study design, year of publication, type of social media, type of evaluations and main outcomes (skills and knowledge measurement, type of skill and knowledge evaluations, delay before assessment, satisfaction and perception evaluations). Data were extracted independently by two authors (AFC and PD), and then their results compared. In case of doubt or disagreement, another author (PM) was engaged.

### 2.4. Quality Analysis

PRISMA guidelines were used for the article research in this review [20]. The quality of the included studies was performed by AFC, PD and MD with the Medical Education Research Quality instrument (MERSQI) for quantitative studies [21,22]. This approach classifies the studies from 0 to 18 points and includes 6 sub-domains on 3 points each (study design, sampling, type of data, validity of evaluation instrument, data analysis, outcomes). A correct methodological quality is usually considered to be above 10 points [23].

## 3. Results

### 3.1. Study Selection 

**Figure 1:**
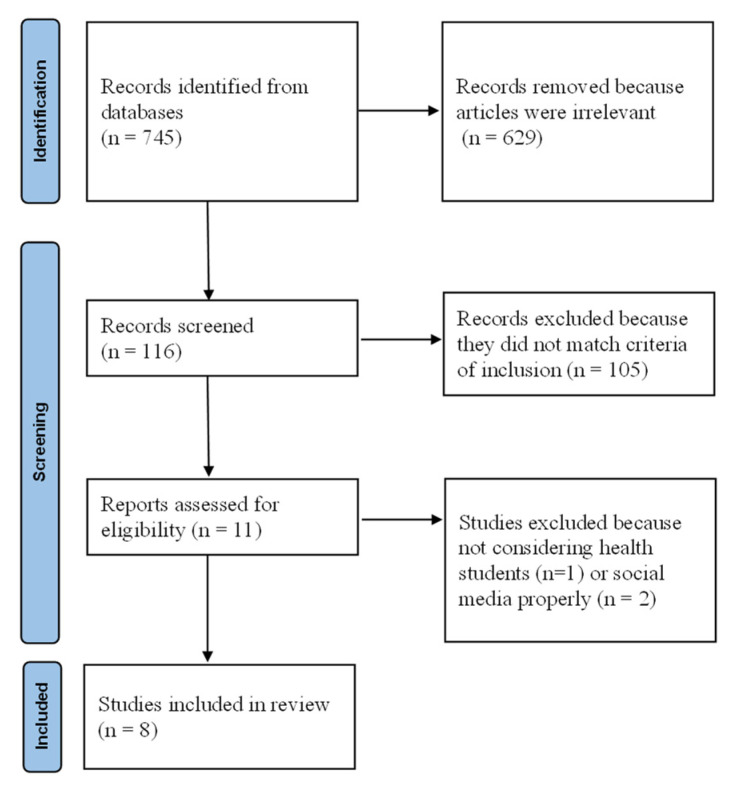
Flow chart of the included studies according to PRISMA guidelines.

The search found 745 results. Out of these records, we kept 116 articles by title. After removing articles that did not meet the criteria of inclusion, 11 articles were assessed for full-text reading. We excluded 3 of them because they did not consider health students or social media properly. We finally included 8 original articles [24,25,26,27,28,29,30,31]. The search strategy for this systematic review is summarized in Figure 1. The articles included participants from 47 to 226 students (Table 2). Quality analysis using the MERSQI of the included studies is reported in Table 3. Their score ranged from 8.0 to 14.5 with a mean of 11.75 ± 2.6 points. 

### 3.2. Demographic Data

Our review assessed 1014 participants. Mean age ranged from 19.9 to 23.4 years, but was not provided in 3 studies [25,26,28]. Seventy percent were female, but sex was not provided in 3 studies [26,28,30] (Table 2). About 54.4% of the participants were medical students [24,25,26,27,30], 20.9% were dental students [29] and 12.3% were students in physical therapy [28].

### 3.3. Subjects Taught and Social Media Used

Various health subjects were considered, from theoretical [25,27,28,30] to practical topics [24,26,29,31] (Table 4).

El-Ali et al. [25] studied the interest of the use of the Radiopaedia.org online platform by third year medical students. They compared the use of Radiopaedia.org in 24 students to a traditional method in radiology education (PDF version of a didactic PowerPoint) in 23 students, for several classic cases of pediatric radiology. Both supports were given at the beginning of the students’ clerkship and were followed by a one hour teaching session in class at the mid-term of the clerkship.

Three studies used the YouTube platform [24,26,30]. Pilieci et al. [24] proposed seven videos about sterile surgical technique to 51 first year medical students compared to one 1.5-h session of skills demonstration in a control group of 63 students. The interventional group had the possibility of watching the videos as many times as they wanted. As to the control group, they were given explanations by a nurse-educator and then could practice under supervision. Karim et al. [26] assessed knee arthrocentesis learning with YouTube by providing a list of standardized videos to 23 pre-clinical medical students [32]. Twenty-four students had traditional teaching by a supervisor and a third group of twenty-four students freely searched for appropriate videos on YouTube. Attardi et al. [30] proposed two videos about anatomy before dissections to 106 first-year medical students in order to compare the anxiety of this group to those of the group of students from the previous university year.

Also, concerning anatomy, Pascoe [28] performed a study comparing 57 physical therapy doctor students who used Snapchat to learn lower limb vascularization in addition to a standard method for 68 students of the previous year who only learned with the standard method. The Snapchat account was only shared with the experimental group and provided still images or short videos with explanations. Each Snapchat story was available for 24 h before being removed and was posted on the same day as the delivery of the course.

In a one-group study of 104 fourth year medical students, Javaeed et al. [27] compared two teaching methods concerning two different medical subjects. Firstly, students learned for one month twenty 1-h lectures using traditional educational strategies on gastrointestinal tract pathology. Then, they had one month of lectures about the cardiovascular system, associated with learning and sharing on different social media: two hours of electronic class on WhatsApp every three days, five high-yield facts on Tweeter every three days, five clinical vignette-based multiple-choice questions on Facebook every three days and discussions on Edmodo with subjects selected by students and quizzes twice a week.

Li et al. [29] studied the impact of WeChat use on 106 dental students studying cariology and comparing to 106 other students of the previous year who had had no access to WeChat. The experimental group had pre-class activities before traditional teaching and could release and discuss images and videos. They also had question–answer sessions with teachers via the social media.

Using Facebook, Chang et al. [31] assessed its interest on 60 paramedical students concerning cultural competence learning compared to a control group of 55 students receiving general information. Facebook documentation included a combination of images, videos, text and polls reflecting cultural competence aspects. Both groups received financial remuneration for completing the tests during the study.

### 3.4. Knowledge Evaluation

Five studies out eight assessed medical knowledge after social media intervention (Table 4). Chang et al. [31] used the nine items of a cultural competence scale to assess knowledge about cultural competence. They found no difference between the group who used Facebook and those who had general information (*p* = 0.325). Pilieci et al. [24] used a 30-item multiple-choice questionnaire to assess knowledge about sterile surgical technique, focusing on scrubbing, growing and gloving, and maintaining sterility. They showed a higher knowledge in the social media group which used YouTube videos compared to the control group, 88.0 ± 1.0% vs. 72.0 ± 1.0% of correct answers (*p* < 0.0001). Concerning knowledge about pediatric radiology, the use of Radiopaedia.org did not show a significant increase in the results for the users compared to the non-users, only a tendency was noticed (74.0% vs. 68.0% of overall correct answers, respectively (*p* = 0.06)) [25]. In their study, Javaeed et al. [27] showed that social media may help one group of students to have higher scores on 100 multiple-choice questions on two separate subjects taught differently (41.8 ± 12.4 for the subject taught without social media vs. 50.8 ± 12.4 for the subject with social media (*p* < 0.001)). The use of WhatsApp was the only social media which provided a positive effect on marks obtained by the students (OR = 4.24; *p* = 0.018), whereas other social media such as Facebook, Edmodo and Twitter had no impact on exam scores. In another study, the use of Snapchat on lower limb blood flow pathway learning provided similar results to traditional learning by comparing two groups of students with a multiple-choice quiz, as both groups had 100% correct answers at the end of the course [28]. One year later, authors reported a low but statistically significant difference between both groups, concerning the difference between the scores at the end of the course and those 12 months later (13.3 ± 14.1% in the control group vs. 9.1 ± 11.5% in the Snapchat group; *p* = 0.04).

### 3.5. Skills Assessment

Only three studies evaluated skills in students [26,29,31] (Table 4). Chang et al. did not show an increase in cultural skills with a self-questionnaire by a Likert-type scale in students using Facebook information compared to other students who had general information (*p* = 0.75) [31]. Karim et al. [26] compared three groups of students on their skill to perform a knee arthrocentesis (groups with classical teaching or standardized videos or searched for videos). They found statistical differences on five parameters: identification of puncture site (*p* = 0.01), wearing gloves (*p* = 0.46), direction of needle insertion (*p* < 0.001) and overall score (*p* < 0.001). For all these parameters, the group “classic teaching” had the highest score. Yet, authors did not provide Bonferroni post-hoc tests to compare the two groups. Li et al. [29] assessed cavity preparation skill levels on a theoretical model in dental students. One group had traditional teaching and the other had pre-teaching preparation via WeChat. The authors found that the group who used WeChat had a higher score on an automatized cavity preparation skill evaluation system about a laser scanning molar model compared to the other group (82.5 ± 6.8 vs. 77.1 ± 5.9, *p* < 0.05). They found statistical differences on five parameters: identification of puncture site (*p* = 0.01), wearing gloves (*p* < 0.001), puncture site sterilization (*p* = 0.046), direction of needle insertion (*p* < 0.001) and overall score (*p* < 0.001).

### 3.6. Student Feedback and Perception

In their study, Attardi et al. [30] aimed to prevent anxiety about anatomical dissection in students with two short YouTube videos. Yet, students who benefited from the videos and those who did not, had similar results concerning trait anxiety (*p* = 0.85) and anatomy state anxiety (*p* = 0.495). With a nine item questionnaire, Li et al. [29] reported a high rate of satisfaction in the experimental group of dental students on cavity preparation skill levels, from 84.9 to 100% of positive responses, but no comparison was performed with the control group. Similarly, Pascoe proposed a satisfaction survey to the social media group about the use of Snapchat on lower limb blood flow pathway learning [28]. Results were mixed. Indeed, 96% of the students found the content of the Snapchat account accurate, 75% were interested in having more courses with the account, 59% were confident in blood flow diagrams and 52% found Snapchat helpful in getting prepared for the exam. Yet only 38% of them found they learned a lot from viewing Snapchat and 27% reported that the social media increased their level of discussion with the class. About the interest of radiology, the use of Radiopaedia.org had no superior effect on the students perception compared to those who had had no access to the platform (*p* = 0.95) [25]. In the study of Pilieci et al. [24] about sterile surgical technique, after knowledge evaluation, both groups received the alternative teaching to complete the follow-up survey. Students reported that the YouTube video method was the most accessible (94%), convenient (82%) and preferentially used to review sterile surgical technique (80%). Yet, the demonstration was considered more helpful to retain knowledge (64%), to scrub in (51%) and easier to complete (40%). Interestingly, no relationship was found between quiz score and learning preference (*p* ≥ 0.1 for all the items assessed) and 97% of the students considered the methods complementary. The use of Facebook formation for cultural competence education showed only an increase of the cultural awareness in the group which used the social media at 3 and 12 months after intervention (*p* = 0.014 and *p* = 0.007, respectively) [31]. Yet, no improvement of self-efficacy perception was shown in the group with Facebook compared to the other group.

## 4. Discussion

The use of social media has increased in health education with several studies pointing out probable advantages concerning short term knowledge but also interactions with other students and teachers, even if their interest has not been definitively established and remains controversial due to possible behavioral drifts [33,34]. Moreover, numerous different types of media are considered which may make it difficult for teachers or students to know which one to choose [35,36,37]. In this review, we have focused our search strategy on studies which could allow a comparison of several methods of learning.

In order to analyze the quality of the included studies, we used the MERSQI. Previously, Reed et al. reported a mean score of 9.95 ± 2.34 on 213 published and peer-reviewed general education studies, which could be considered the medium quality score [21]. After analysis of the quality of our included articles, we found a mean score of 11.7 ± 2.6, which is higher than those of Reed et al. [21], but also much higher than previous findings on social media and education, with mean scores from 8.8 ± 3.3 to 9.5 ± 2.0 [38,39,40]. These differences are certainly due to the high specificity of our selection criteria that allowed us to assess only comparative studies of several learning methods, with high quality methodology for the most part, even if we included two studies with lower scores of 8 and 8.5 [27,28].

Concerning knowledge evaluation, we found that the use of social media may have a rather positive impact from a short-term point of view. Indeed, Pilieci et al. reported a significantly higher rate of knowledge about sterile surgical technique after using YouTube videos compared to traditional teaching (*p* < 0.0001) and El-Ali et al. found a trend toward better knowledge about pediatric radiology in the social media group using both Radiopaedia.org and traditional learning. (*p* = 0.06) [24,25]. Consistently, Snapchat associated with standard instruction could have a positive impact on long term knowledge about anatomy [28]. Indeed, at the end of the teaching, the Snapchat group and the traditional learning group had completed the test without error, but the group that used Snapchat seemed to have better recall one year later. However, these findings should be taken cautiously because despite being statistically significant, the difference between the two groups was only 4%. Interestingly, Javaeed et al. found that results may vary according to the social media activity with a better impact in case of more active participation with WhatsApp compared to other social media used more passively [27]. This may be consistent with the results of Chang et al. who used Facebook and found no improvement in knowledge compared to classic information about competence education, both in the medium and long term [31]. However, this last study did not clearly define what the control group performed and what their “information” was.

Conversely, social media seemed to have a more contrasting effect on skills. Indeed, concerning arthrocentesis learning, the group which had a traditional explanation and demonstration showed better results on all the analyzed criteria compared to those who had only YouTube tutorials [26]. Chang et al. found no difference in skills related to cultural competence when using exclusively Facebook [31]. In these two studies, the interventional and the control groups had strictly different teaching and the interventional group had only social media learning. In contrast, Li et al. reported higher scores for dental students who studied with WeChat in addition to traditional learning concerning buccal cavity preparation, but these results may be explained by the fact that both groups had the same traditional teaching [29]. So, WeChat learning could be considered as an interesting complementary tool. Furthermore, the results of these three studies are limited because skills were not assessed in a context of care with patients but on a knee anatomical model [26], a buccal computer reconstruction [29] or with a Likert-type self-questionnaire [31]. 

Literature usually reports a positive perception of students on the use of social media [41,42,43]. However, our review reports more contrasting results. Indeed, YouTube videos failed to reduce anxiety of students about anatomy dissection [30], whereas they were positively considered by students about surgical sterile technique (accessibility, practicality, revisions), even if demonstrations were considered better to retain knowledge and to acquire scrubbing technique [24]. Furthermore, health students did not report more self-efficacy on cultural competence when using Facebook [31].

According to these findings, we can assume that social media may have a positive impact on learning in health students but more as an adjunct to classical teaching rather than as an alternative one. Indeed, the positive effects were shown mainly when both methods were used together [25,27,29]. Yet, we can point out that these results may be due to the fact that the students had more learning hours than the others. Thus, the positive effects could have been linked to this extra work and not specifically due to the type of method used. Moreover, the methods of student evaluation were mainly locally created by the authors and are rarely tools accessible and previously evaluated before their use, which may raise questions about their relevance and reproducibility. Finally, it is important to keep in mind that social media may also have a negative impact on students. Indeed, these young people may be fragile and may be at risk of cyberbullying or social media addiction in the more severe cases [44,45], but social media could also be responsible for an increase in their distractibility and have consequences on concentration [46].

Finally, our review has also limitations. Indeed, comparisons between studies remain difficult because the authors studied different types of knowledge or skills, different criteria of evaluation or different social media. Nevertheless, we tried to find common criteria of evaluation and describe precisely the outcomes of each study so as to give the reader an overview of this growing topic. Furthermore, the term “social media” could be questionable as there is fast and multimodal development in this area, and there is no current consensus on its definition [10]. So, to include YouTube and Radiopaedia.org could be debated as they are a video distribution platform and an online radiologic database. Yet, YouTube enables interactions between users by liking, posting content, replying, sharing and commenting on each digital material. In the same way, Radiopaedi.org is an online platform which enables clinicians and students to have access to numerous radiology references, to create new radiological cases and to share them with the medical community [47]. This is totally in line with the definition of social media used in this work [16,17]. Besides, we did not perform a meta-analysis in order to avoid misleading because of the heterogeneity of the included studies regarding interventions, outcomes, and subjects. As we were dealing with different teaching methods evaluated differently, we chose to consider the studies separately.

## 5. Conclusions

The use of social media has developed considerably in health studies, this development is an important offering, however, is sometimes difficult to assess. It seems to have a relatively positive impact on knowledge acquisition with, however, possible variations depending on the tools used. The use of social media seems more questionable concerning skills, with rather disappointing results in terms of added value and a lack of evaluation in care situations. Social media are more a complementary learning method than a complete alternative to traditional courses and it is not excluded that their effects could lead to an increase of working time for the students. In addition, social media may also have negative effects on students such as screen addiction or more frequent lapses of concentration. In the future, the impact on patient care should be assessed to better analyze the possible contribution of social media on the improvement of medical practices.

## Figures and Tables

**Table 1:** Search terms used in PubMed database.

Query	Search Term
#1	“Social media” [All Fields] OR “Social network” [All Fields]
#2	“Student” [All Fields] OR “learning” [All Fields] OR “teaching” [All Fields]
#3	“medical” [All Fields] OR “dental” OR “nurse” [All Fields] OR “midwife” [All Fields]
#4	#1 AND #2 AND #3

**Table 2:** Demographic data of the included studies.

Studies	Total Participants (*n*) (Experimental + Controls)	Mean Age (Years)	Gender (M/F)	Type and Level of the Students	Subjects Taught	Type of Social Media
Chang et al. 2017 [31]	115 (60 + 55)	23	27/87	Students from professional nursing, pharmacy and nutrition programs	Cultural competence education	Facebook
Pilieci et al. 2018 [24]	114 (51 + 63)	22.9	43/71	First year medical students	Sterile surgical technique	YouTube
El-Ali et al. 2019 [25]	47 (24 + 23)	?	22/25	Third year medical students	Pediatric radiology	Radiopaedia.org
Karim et al. 2020 [26]	71 (47 + 24)	?	?	Preclinical medical students	Knee arthrocentesis	YouTube
Javaeed et al. 2020 [27]	104	22.9	29/75	Fourth year MBBS students	Gastrointestinal tract pathology and cardiovascular system pathology	Facebook, Edmodo, Twitter and WhatsApp
Pascoe, 2021 [28]	125 (57 + 68)	?	?	Entry-level doctor of physical therapy	Anatomy of lower limb vascularization	Snapchat
Li et al. 2021 [29]	212 (106 + 106)	19.9	54/158	Dental students	Cariology	WeChat
Attardi et al. 2021 [30]	226 (106/120)	23.4	?	First year medical students	Anatomy	YouTube

Abbreviations: M: male; F: female; MBBS: Bachelor of Medicine and Surgery.

**Table 3:** Evaluation of the studies quality using the Medical Education Research Quality instrument (MERSQI).

Studies	Design	JCR-WOS *	Scopus Highest Percentile *	MERSQI Items	Study Design /3	Sampling /3	Type of Data /3	Validity of Evaluation Instrument /3	Data Analysis /3	Outcomes /3	Total Score /18
Chang et al. 2017 [31]	RCS not blinded	2.067	93%		3	2.5	3	2	2	2	14.5
Pilieci et al. 2018 [24]	RCS not blinded	1.921	72%		3	2	3	0	2	1.5	11.5
El-Ali et al. 2019 [25]	RCS not blinded	4.268	64%		3	2	3	1	2	1	12
Karim et al. 2020 [26]	RCS not blinded	N/A	75%		3	2	3	1	2	2	13
Javaeed et al. 2020 [27]	One group crossover	N/A	76%		1	0.5	3	0	2	1.5	8
Pascoe, 2021 [28]	Comparative trial between a prospective group and a retrospective one	N/A	N/A		1	2	1	2	1	1.5	8.5
Li et al. 2021 [29]	Prospective comparative study	5.428	85%		2	2	3	1	1	1.5	10.5
Attardi et al. 2021 [30]	Prospective comparative study	5.958	96%		2	2	3	3	3	1	14

Abbreviation: RCS = randomized controlled study; JCR = Journal Citation Reports ™; Web of Sciences; * information for the year of publication, except for 2021 for which the year 2020 was used; N/A = not applicable.

**Table 4:** Type of interventional method used, type of evaluation and main outcomes.

Studies	Social Media Group	Control Group	Skills and Knowledge Measurement	Type of Skill and Knowledge Evaluations	Delay before Assessment	Satisfaction and Perception Evaluations
Chang et al. 2017 [31]	12 topics using videos, text and images	General information	Cultural competencies	9 true/false knowledge items of the CCS 19 Likert-type skill items	Before, 3 and 12 months after the program	6 Likert-type awareness items 15 Likert-type self-efficacy items
Pilieci et al. 2018 [24]	7 videos from 2 to 6 minutes	One 1.5 h-session of skill demonstrations	Knowledge about: - Scrubbing - Gowning and gloving - Maintaining sterility	30-items multiple-choice quiz	Early after the formation (no exact delay provided)	23-item questionnaire on preferred educational format
El-Ali et al. 2019 [25]	Access to Radiopaedia playlist of pediatric radiology before 1-h in-class interactive teaching	PDF version of a didactic PowerPoint before 1-h in-class interactive teaching	Important basic concepts in pediatric radiology	10 radiology knowledge-based questions	At the beginning of the clerkship and 1 month later	6 questions regarding student opinion of radiology and their own ability to interpret radiology studies
Karim et al. 2020 [26]	Videos provided with links or self-searched	Supervisor-led session	Skills about 7 steps for performing knee arthrocentesis on a knee aspiration model	Student performances assessed by an examiner with checklist/grading sheet	10 minutes	None
Javaeed et al. 2020 [27]	20 lectures on gastrointestinal tract and 20 lectures on cardiovascular system associated with learning on WhatsApp, Twitter, Facebook and Edmodo	None	Knowledge about gastrointestinal tract and cardiovascular system pathologies	100 multiple choice questions for each subject	The day after completion of the gastrointestinal sessions and 3 days after the completion of cardiovascular sessions	None
Pascoe, 2021 [28]	Videos and still images and standard anatomy instruction	Standard anatomy instruction	Knowledge about blood flow pathways of the lower limb	Multiple-choice quiz	At the end of the course and at 12 months	12-item survey about satisfaction
Li et al. 2021 [29]	Pictures and short videos before traditional teaching	Traditional teaching	Automatized comparison between a prepared tooth and an ideal one	Cavity preparation skill levels (on theoretical model)	At the end of the training course	Evaluation of the experimental group with a 9-item questionnaire
Attardi et al. 2021 [30]	Two short videos prior to anatomical dissection	Usual courses	None	None	Prior to cadaveric anatomy lessons	State–trait anxiety inventory scale
